# Vertebral morphology in the tail-whipping common thresher shark, *Alopias vulpinus*

**DOI:** 10.1098/rsos.231473

**Published:** 2024-01-03

**Authors:** Jamie L. Knaub, Michelle Passerotti, Lisa J. Natanson, Tricia Meredith, Marianne Porter

**Affiliations:** ^1^ Department of Biological Sciences, Florida Atlantic University, Boca Raton, FL, USA; ^2^ FAU Lab Schools, Florida Atlantic University, Boca Raton, FL, USA; ^3^ Apex Predators Program, Northeast Fisheries Science Center, National Oceanic and Atmospheric Administration (NOAA), Narragansett, RI, USA

**Keywords:** centra, mineralized cartilage, micro-computed tomography, microstructure

## Abstract

Thresher sharks (*Alopias* spp.) are characterized by an elongated, scythe-like caudal fin that is used in tail-whipping, a behaviour where the tail is thrown overhead to stun prey. Tail-whipping is performed via extreme dorsoventral bending of the vertebral column, and is dramatically different from lateral oscillatory motion used for swimming. Previous work has examined thresher shark vertebral morphology and mechanical properties, but in the context of swimming loads. Our goal was to assess centra morphometrics and microarchitecture for variations that may support extreme dorsoventral bending. We examined anterior and posterior body vertebrae from an embryo, five juvenile, and four adult thresher sharks using micro-computed tomography. We used principal component and landmark analyses to examine variables influencing vertebral morphology and mineral arrangement, respectively. We found that morphology and microstructure significantly varied across body regions and ontogeny. We hypothesize that anterior body vertebrae increase stability, while posterior body vertebrae support the caudal fin. Vertebral size and quantity of mineral structures (lamellae and nodes) increased across ontogeny, suggesting vertebrae adapt over development to support a larger body and tail. Based on our results, we hypothesize that thresher shark vertebrae vary in morphometrics and mineralization (amount and arrangement) supporting the mechanical needs for tail-whipping.

## 1. Introduction

Family Alopiidae, the thresher sharks, is a small group within order Lamniformes and comprises three species: the pelagic thresher (*Alopias pelagicus*)*,* the bigeye thresher (*Alopias superciliosus*)*,* and the common thresher (*Alopias vulpinus*) [1–3]. Perhaps the most striking physical characteristic of thresher sharks is the caudal fin's scythe-like upper lobe, which is nearly the length of the body [1–3]. The caudal fin is used in a tail-whipping behaviour to strike, disorient, and corral prey such as small to medium schooling fishes [1,4–8]. This tail-whipping movement dramatically differs from the lateral undulations produced by the body during swimming. The vertebral column, the main body axis, may have morphological adaptations to withstand the extreme axial bending during the thresher shark tail-whipping behaviour [8].

The tail-whipping strategy was first speculated following visual accounts of the behaviour of tail-hooked thresher sharks caught on longline and trolling lures [9–13]. Juveniles and adults of both sexes of common thresher sharks have been recorded using the upper lobe of the caudal fin to strike tethered bait or prey items [4,8]. More recently, video footage of hunting events by the pelagic threshers documented both sideways and overhead tail-whips [8].

**Figure 1.**
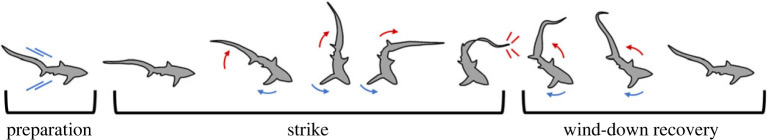
Thresher shark overhead tail-whipping behaviour. Overhead tail slaps begin in the preparation phase by lunging towards targeted prey. The strike phase begins by lowering the head and flexing the body ventrally. By adducting the pectoral fins (blue arrows), the posterior body is raised overhead, and dorsal extension sends the caudal fin (red arrows) forward, whipping the prey. The wind-down recovery phase consists of the shark returning to swimming posture and is followed by the prey collection phase (not pictured). Graphic adapted from behavioural illustrations of pelagic thresher sharks [8].

Tail-whipping kinematics and related behaviour of the pelagic thresher shark from the Philippines were quantified from underwater video observations [8]. Below, we summarize thresher shark tail-whipping behaviour described from observations and video recordings by Oliver *et al.* [8]. Overhead tail whips are described as a trebuchet, a specific type of catapult, that uses a counterweight to throw an object from a sling at the end of a long beam. Thresher shark tail whipping consists of four phases: preparation, strike, wind-down recovery, and prey collection (figure 1) [8]. Characteristic behaviours of the preparation phase include horizontal lunging towards prey items, while tail strikes are initiated by lowering the snout and ventral flexion of the body. The pectoral fins are adducted, and the posterior body region is rapidly raised resulting in a braking effect that slows the forward lunge. Dorsal extension of the trunk accelerates the upper lobe of the caudal fin cranially, whipping it overhead towards prey items and terminating in a whip. The apex of the whip occurs above the dorsal fin and in several hunting events, was documented with the appearance of bubbles, believed to be caused by cavitation, dissolved gas diffusing out of the water column. The wind-down phase begins when the tail fully extends, terminating above the snout, and this is followed by the head, peduncle, and pectoral fins returning to the initial swimming posture. Prey collection occurs when the shark pursues the stunned fish. Sideways whips, observed only after successful overhead strikes, consist of the shark aligning parallel to the targeted prey items and exerting the caudal body and fin horizontally to strike in a lateral direction [8].

The vertebral column is comprised of cartilaginous centra separated by intervertebral capsules, which experience millions of bending cycles over a lifetime [14]. During swimming, oscillatory waves travel down the body and subject vertebrae to lateral tension and compression forces over successive tailbeats. These forces alternate with side-to-side tail movements, and the mineralized structures within vertebrae have adapted to meet the mechanical demands of oscillatory loading [15]. Deformation (strain) within centra during swimming permits greater total dislocation of the vertebral column [16,17]. Mechanically, greater deformation along the column allows cartilaginous vertebrae to have increased elastic energy storage compared to bony fish, and vertebrae can be used as a biological spring or brake system depending on tail beat frequency [16,17].

Within shark centra, the amount and arrangement of mineral is known to vary both intra- and interspecifically, and in part determines the material properties of centra [18–23]. When mechanically compressed to failure, shark vertebrae exhibit stiffness (ability to resist compression) similar to mammalian trabecular bone [19,20,24]. At biologically relevant compression strains, stiffness and toughness (ability to absorb energy) are positively correlated, and these two mechanical properties operate in concert to support lateral oscillations during swimming [25]. The mechanical properties of lamniform shark centra vary along the body and across ontogeny; posterior body centra and from juvenile sharks are stiffer and tougher than anterior body centra and from adult sharks, respectively [25]. The observed mechanical behaviour of shark centra may be attributed to variation in microarchitecture across body regions and ontogeny.

**Figure 2.**
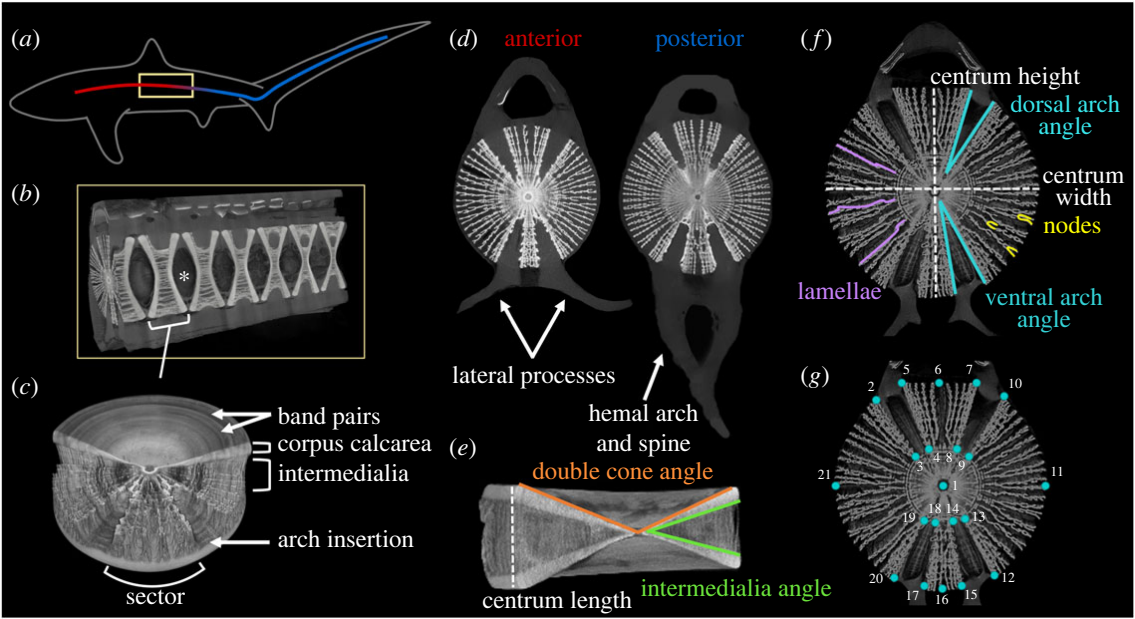
Thresher shark vertebral macro- and microstructure. (*a*) Whole body diagram of a vertebral column (gradient line) within a thresher shark. Vertebrae are cylindrical and a consecutive section outlined in yellow is shown in (*b*). (*b*) Cutaway of vertebrae; the frontmost is the transverse midplane section displaying the four sectors of radiating lamellae and gaps in mineralization for the arch insertions. Centra are separated by an intervertebral joint (an example joint is depicted by an asterisk). (*c*) Three-quarter view of whole centrum; band pairs are visible on the dorsal centrum face. A cutout displays lamellae radiating from the centre of the centrum and forming the intermedialia that stretches between the corpus calcarea (double cone structure). (*d*) Transverse view of thresher shark anterior and posterior body vertebrae along the vertebral column. In anterior body vertebrae, lateral processes project outwards, whereas posterior body vertebrae have a hemal arch and spine that project ventrally. (*e*,*f*) Morphometrics and mineral architecture measured. A sagittal and transverse cross section showing variables quantified: centrum height, width, and length, lamellae count, node count, dorsal and ventral arch angles, intermedialia angles, and double cone angles. (*g*) Transverse cross section showing 21 digitized landmarks (points in cyan, labels in white) used for geometric morphometric analysis.

A single centrum is cylindrical due to the hourglass-shaped corpus calcarea, or double cone structure (figure 2) [15,21,22,24,26,27]. In lamniform sharks, radiating plates (lamellae), which can have bifurcating nodes, stretch between the arms of the corpus calcarea and form the intermedialia (figure 2) [15,21,26,28]. In the transverse midplane, the intermedialia form four sectors; two large groups of lamellae project laterally and are separated from the smaller dorsal and ventral sectors by gaps in mineralization where the vertebral arches insert (figure 2) [15,21]. Growth of vertebral centra, correlated with shark girth, varies along the body axis [15,26]. During growth, band pairs (visible concentric rings; figure 2) are deposited within the double cone wall and are evident in lamellae of lamniform shark centra, yet the structural role of these rings within the calcified architecture remains unknown [15,26].

Vertebral architecture has been previously described using micro-computed tomography (CT) imaging of one common thresher shark centrum from an unknown body location [15]. In this specimen, lamellae in lateral regions of intermedialia bifurcated (nodes), whereas in dorsal–ventral regions and surrounding the mineralization gaps, the lamellae branched and re-integrated [15]. In comparison to other lamniform sharks (white and shortfin mako) the thresher shark centrum had the largest cone angles, smallest gaps between mineralized sectors, and the thickest inner cone wall (proximal to the focus) and thinnest outer wall (adjacent to the edge) [15]. Additionally, the common thresher shark mineral density of the lamellae was comparable to that of the cone wall, while the intermedialia of carcharhiniform shark vertebrae had significantly less mineral than the cone wall [15]. A visual comparison of lamniform shark centra highlights the variable morphology of thresher shark vertebrae, specifically the quantity of lamellae and nodes, that may contribute to the tail-whipping behaviour (electronic supplementary material, figure S1).

The first goal of this study is to quantify vertebral morphology and internal mineral architecture across body regions and ontogeny to assess morphological variations that may facilitate the tail-whipping behaviour of thresher sharks. To investigate vertebral morphology, we measured centrum morphometrics (height, width, length) along the vertebral column from ten common thresher sharks across a range of sizes from an embryo to large adults. We hypothesized that thresher shark centra would be larger in the anterior body region then become shorter while height and width remain the same in the posterior body region, as has been previously described [26,29]. Second, we used micro-CT scanning to image the internal mineral architecture of each centrum. We quantified the number of mineral structures (lamellae and nodes) and centrum morphology (arch insertion angles, intermedialia angles, double cone angles, and volume of mineralized cartilage). Additionally, we used two-dimensional (2D) geometric morphometric analysis to examine variation in the spatial distribution of mineral structures along the vertebral column. We hypothesized that posterior body centra will have a greater quantity of lamellae and nodes, smaller arch insertion and intermedialia angles, and greater double cone angles and centrum volume to support the mechanical demands of tail-whipping (extreme axial bending) in thresher sharks. Finally, we investigated the scaling relationship between caudal fin length and shark body length. We hypothesized a linear relationship, where the caudal fin will grow proportionally with the rest of the body across ontogenetic stages.

## 2. Methods

### 2.1. Specimens

In this study, we examined vertebrae from ten common thresher sharks (*Alopias vulpinus*) ranging from an embryo (fork length (FL): 61 cm) to large adults (FL: 241.9 cm; table 1). Specimens were collected between 2014 and 2018 by the National Marine Fisheries Service, and one specimen was collected in 2022 (FL: 172.0 cm) from SeaWorld San Diego, Coronado, CA (SHK-EFP-17-01). Sharks were collected following strandings, research surveys, and recreational fishing events along the United States Atlantic coast and Pacific coast for the one California specimen. For this project, we received segments of consecutive vertebrae from anterior and posterior body regions that were frozen and stored in a −20°C freezer.

**Table 1.** Specimen vertebral samples and ontogenetic status determined by body measurements.

fork length (FL, cm)	total length (TL, cm)	sex	ontogenetic status	number of vertebrae	body region
61.0	120.8	M	embryo	7	both
159.5	—	F	juvenile	6	both
162.9	299.5	F	juvenile	5	anterior
172.0	273.0	F	juvenile	19	both
176.7	300.0	M	juvenile	7	anterior
180.8	328.0	F	juvenile	6	anterior
208.0	—	M	adult	19	posterior
209.9	408.0	M	adult	5	anterior
229.0	—	F	adult	6	both
241.9	—	F	adult	5	anterior
10 sharks				85 vertebrae	

Sharks for which total length data were unavailable are denoted with a —.

We compiled life history and morphometric data for each shark including sex and FL (cm), which were measured as per Natanson *et al*. [30]. FL is measured from the tip of the nose to the fork in the caudal fin (table 1). For some specimens, total length (TL, cm) was measured as the distance from the tip of the nose to the end of the caudal fin upper lobe, and those data are included here (table 1). To assess vertebral morphology in an ontogenetic context, we used the previously published data on median length at maturity for this species: 216 cm FL and 188 cm FL for females and males, respectively [31]. On this basis, we had one embryo, five juvenile (immature) specimens and four adult (mature) thresher sharks in this study (table 1).

Along the vertebral column, we classified each centrum as anterior or posterior body based on the morphology of the ventral processes and location along the body. Anterior body vertebrae have processes that project laterally from the ventral portion of each centrum. In the caudal portion of the abdominal cavity, the processes join to form the hemal arch and spine. All anterior body vertebrae were sampled from underneath or cranial to the first dorsal fin, and posterior body vertebrae were sampled from the precaudal pit (longitudinal notches on the caudal peduncle just prior to the caudal fin insertion; figure 2*d*).

### 2.2. Micro-computed tomography

Prior to imaging, vertebral samples were thawed and measured with digital calipers in the following dimensions: greatest height (measured dorsoventrally, mm), greatest width (measured laterally, mm), and greatest length (measured in the cranial–caudal direction, mm; figure 2*e*,*f*). For CT scanning, we oriented consecutive vertebrae vertically (to match the upright cylindrical geometry of the scanned area) in a plastic canister with polyurethane foam for stabilization prior to sealing them with cling wrap. We imaged samples from nine animals with a Bruker SkyScan 1173 system (Kontich, Belgium) at 120 kV (kilovolts), 60 µA (amperage, X-ray intensity), between 18.2 and 32.0 µm voxel size, and 1.0 mm aluminium filter. We reconstructed images using Bruker Nrecon software and created a three-dimensional (3D) rendering of vertebral samples with Bruker CTVox software. Mineral structures in centra from embryos are not well defined and thus we excluded the embryo vertebrae from the mineral structure counts and principal components analysis (PCA), but included them in other variables of centra morphology as described below.

The Bruker SkyScan was out of commission for some weeks for service; therefore 19 vertebrae (6 anterior and 13 posterior body) from one thresher shark (FL: 172.0 cm) were CT scanned at the University of Florida's Nanoscale Research Facility using a GE Phoenix V|Tome|X system (GE Measurement & Control, Boston, Massachusetts, USA). The extracted vertebral samples were imaged in two separate multi-scans (anterior and posterior body segments) at a 35 µm voxel size, 120 kV, 250 µA, and 0.1 mm copper filter. We reconstructed raw scan files using datos|x software and created 3D renderings using Volume Graphics (myVGL) software.

### 2.3. Vertebral structure and morphometrics

Using Bruker CTVox 3D visualization software, we captured three replicate images for centra in the transverse plane at the cross-section of the double cone apex, and in the sagittal plane at the cross-section of the intermedialia. We assigned replicate images a blind ID to reduce unintentional bias during data collection. Using ImageJ, we quantified arch angles, intermedialia angles, and double cone angles for ten sharks (figure 2*e*,*f*) [32]. We quantified the number of mineral structures (lamellae and nodes) in all individuals except the embryo from visual counts performed by one researcher. Counts and angle measurements were averaged across replicates for each centrum.

To compare spatial locations of mineral structures within vertebrae across body regions and individuals, we used a 2D geometric morphometric analysis on the middle transverse slice (transverse bisection through the focus) from each centrum. We digitized 21 landmarks to map the mineralization layout using tpsDig software (figure 2*g*) [33]. We used the landmark coordinates in a 2D geometric morphometric assessment using MorphoJ software and performed a Procrustes fit to generate a covariance matrix.

For each centrum, we quantified volume (mm^3^) by segmenting voxels of mineralized cartilage using SlicerMorph, an extension to 3D Slicer [34]. We created segmentations using the *Segment Editor* module, which allowed thresholding to capture only the mineralized cartilage. We used *Remove Islands* and *Fill Holes* segmentation features on an individual basis depending on thresholding appearance in 3D views. For all segmentations, we used the *Smoothing* tool prior to extracting the volume (mm^3^) using the *Segmentation Statistics* module. We divided the centra volume by fork length to scale with specimen size.

### 2.4. Statistical analysis

All ten common thresher sharks examined in this study had FL data, and six individuals (the embryo, four juveniles, and one adult) had TL data available (table 1). For five of those individuals, we subtracted FL from TL to determine caudal fin length (CFL). We excluded the embryo when calculating the regression because it was under development and had not yet reached postnatal proportions. We then divided CFL by FL, which is a ratio of the size of tail relative to the size of the body. We then plotted a regression with the CFL:FL ratio against TL to examine the scaling of the body and caudal fin.

We analysed data using JMP 10 (SAS Institute Inc.) and Rstudio software [35]. Lamellae count, intermedialia angle, and double cone angle met the normality and homogeneity assumptions. We used a two-way ANOVA to analyse the effect of body region (anterior and posterior body), ontogenetic status (juvenile and adult, determined by fork length), and an interaction term (body region × ontogenetic status) on lamellae count, intermedialia angle, and double cone angle. We performed Tukey's HSD *post hoc* tests to identify significant differences among effects. Centra morphometrics and volume were standardized to individual shark fork length. Node count, dorsal arch angle, ventral arch angle, centra morphometrics, and centra volume did not meet assumptions and failed to meet test assumptions following transformations. These variables were assessed using Wilcoxon/Kruskal–Wallis rank sums tests and non-parametric comparisons. For non-parametric tests, centra were classified into one of four levels: Anterior-Juvenile, Anterior-Adult, Posterior-Juvenile, and Posterior-Adult.

To further examine structural variation in postnatal individuals (*N* = 9), we used a PCA (function *prcomp*) with body region and ontogenetic status as the main grouping effects. We used an ANOVA to assess significance of body region and ontogenetic grouping on resulting PCA axes. To assess 2D landmark data, we used the Procrustes fit covariance matrix to generate a PCA examining geometric variation of mineral landmarks on the transverse cross-section. We used a Procrustes ANOVA to analyse the effect of body region on spatial landmarks and shape in MorphoJ software [36,37].

## 3. Results

We plotted a regression of the CFL:FL ratio against TL for five sharks. A fitted linear regression showed slight positive allometry for the five postnatal individuals (CFL:FL = 0.0022TL + 0.0563) with a high correlation coefficient (*R*^2^ = 0.720; electronic supplementary material, figure S2). The increase in CFL:FL ratio across ontogeny (from 0.587 in the smallest postnatal shark to 0.944 in the adult) suggests the caudal fin is proportionally, relative to body size, longer in adults than juveniles. In the adult specimen, the CFL:FL ratio of 0.944 indicates that the body and caudal fin are nearly the same size, while juvenile shark bodies are larger than the caudal fin.

### 3.1. Vertebral structure

**Figure 3.**
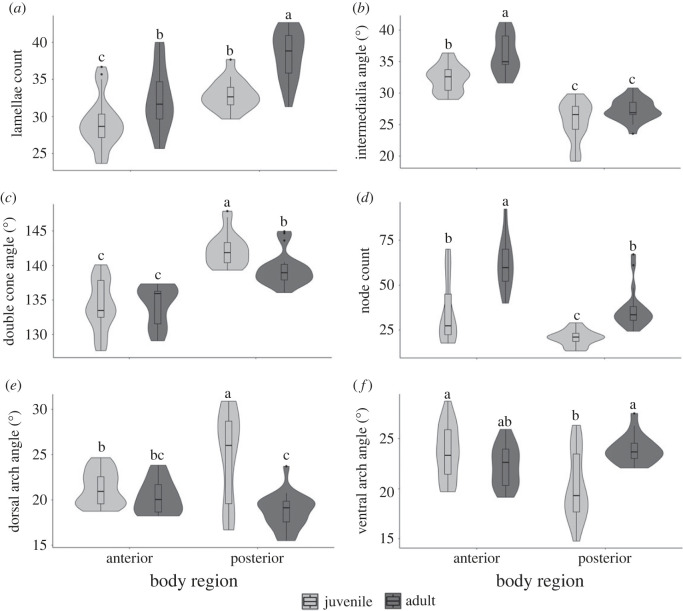
Mineral structures and centra morphology across body regions and ontogenetic status. The width of each violin indicates distribution, a boxplot within each violin depicts quartile ranges, and the median value is shown as a horizontal line. Tukey *post hoc* results indicate significant differences between groups. (*a*) Posterior body centra had higher counts of lamellae, and the quantity of lamellae increased across ontogeny. (*b*) Anterior body centra had larger intermedialia angles compared to posterior body centra, and the angle increased over ontogeny. (*c*) Posterior body centra had larger double cone angles than anterior body centra. The angle of the double cone increased in anterior body centra across ontogeny but decreased in adult posterior body centra. (*d*) Anterior body centra had higher counts of nodes and the quantity of nodes increased across ontogeny. (*e*) Anterior body centra had comparable dorsal arch angles across ontogenetic stages but posterior body centra had larger angles in juveniles compared to adults. (*f*) Ventral arch angles were comparable in anterior body centra across ontogenetic stages but adult centra had larger angles in the posterior body region.

A two-way ANOVA examining lamellae number was significant (*F*_3,77_ = 31.985; *p* < 0.0001; electronic supplementary material, table S1). Both body region (anterior and posterior, *F*_1,1_ = 40.445; *p* ≤ 0.0001) and ontogenetic status (*F*_1,1_ = 30.530; *p* ≤ 0.0001) were significant effects, and the body region by ontogenetic status interaction was not significant (*F*_1,1_ = 2.161; *p* = 0.146). Tukey *post hoc* tests showed that centra from the posterior body of adult sharks had the greatest lamellae counts. Anterior adult and posterior juvenile centra had comparable counts and anterior juvenile centra had the fewest lamellae (electronic supplementary material, table S2; figure 3*a*).

The two-way ANOVA examining intermedialia angle was significant (*F*_3,84_ = 65.778; *p* < 0.0001) and both body region (*F*_1,1_ = 194.713; *p* < 0.0001) and ontogenetic status (*F*_1,1_ = 23.694; *p* = 0.0001) were significant effects, but the interaction was not significant (*F*_1,1_ = 3.156; *p* = 0.079; electronic supplementary material, table S1). Larger intermedialia angles were observed in anterior body centra and adult individuals (electronic supplementary material, table S2; figure 3*b*).

A two-way ANOVA examining double cone angle was significant (*F*_3,84_ = 38.733; *p* < 0.0001). Body region was the only significant effect (*F*_1,1_ = 98.233; *p* < 0.0001); posterior body centra had larger double cone angles (electronic supplementary material, table S2; figure 3*c*). Ontogenetic status was not a significant effect in the ANOVA (*F*_1,1_ = 3.788; *p* = 0.055). The interaction term (body region × ontogenetic status) was significant (*F*_1,1_ = 5.501; *p* = 0.022); anterior body centra did not have significantly different double cone angles between juvenile and adult sharks, but posterior body centra had significantly larger double cone angles in juvenile sharks (figure 3*c*).

Wilcoxon/Kruskal–Wallis rank sum tests were significant for centra morphometrics. Centrum width (*H* = 17.380, 3 d.f., *p* = 0.0006) and height (*H* = 39.838, 3 d.f., *p* < 0.0001) significantly varied across levels (anterior juvenile, anterior adult, posterior juvenile, and posterior adult; electronic supplementary material, table S3). Centra were widest in adult sharks and in the anterior body. Centrum height was greatest in adult sharks, and anterior body centra were taller than posterior body centra. Centrum length was significant across all levels (*H* = 68.423, 3 d.f., *p* < 0.0001) and they were shorter in the posterior body region and juvenile sharks. Centrum volume significantly varied across levels (*H* = 67.235, 3 d.f., *p* < 0.0001); adult shark centra did not differ significantly across body regions but had significantly larger volumes than juvenile sharks. In juvenile sharks, anterior body centra had significantly greater volumes than posterior body centra.

Wilcoxon/Kruskal–Wallis rank sum tests were significant for mineral architecture variables. Node counts were significantly different across levels (*H* = 40.088, 3 d.f., *p* < 0.0001; figure 3*d*; electronic supplementary material, table S3); a greater number of nodes were observed in the anterior body region and adult sharks. Arch angles were significantly different across levels. Dorsal arches (*H* = 22.786, 3 d.f., *p* < 0.0001; figure 3*e*) were larger in the posterior body region, and in juvenile individuals. Ventral arches (*H* = 16.002, 3 d.f., *p* = 0.0011; figure 3*f*) were larger in the anterior body region and in adult sharks.

### 3.2. Mineral architecture principal component analysis

**Figure 4.**
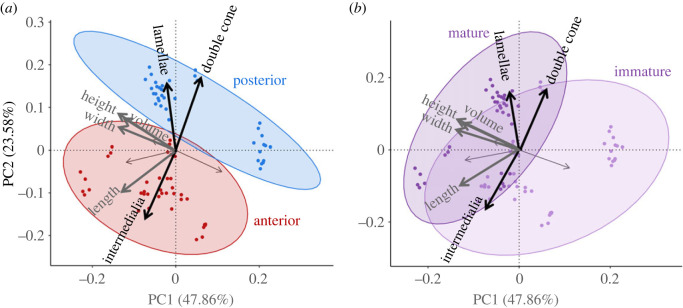
Mineral architecture PCAs. PC1 explains 47.86% variation and PC2 explains 23.58% (71.44% across two axes). Ellipses are drawn as 95% confidence intervals for each grouping. Highest loading variables on PC1 and PC2 are shown in bold grey and black arrows respectively. (*a*) PCA across body regions. Anterior and posterior body centra occupied opposing regions on both PC1 and PC2. Anterior body centra occupied lower values on both axes while posterior body centra had higher PC values on PC1 and PC2. (*b*) PCA across ontogenetic status. Juvenile and adult shark distribution differed mostly on PC1 with overlap on PC2.

Mineral architecture data and centra morphometrics from nine sharks were used in the PCA. The embryo was not incorporated in this analysis because it was not well mineralized, and no distinction could be made between lamellae and nodes. PC1 and PC2 accounted for 47.86% and 23.58% of the variation, respectively (71.44% variation explained across two axes; electronic supplementary material, table S4). Main loadings on PC1 were centrum width, height, length, and volume (electronic supplementary material, table S5). Main loadings on PC2 were double cone angle, intermedialia angle, and lamellae count (electronic supplementary material, table S5). We grouped ellipses by body region and ontogenetic status using 95% confidence intervals (figure 4). PC1 negatively correlated with centrum morphometrics and volume. The posterior body region ellipse occupied higher scores on PC1, aligning with smaller centrum size and volume compared to anterior body centra that were larger (figure 4*a*). PC2 positively correlated with lamellae count and double cone angle. Posterior body centra were distributed at higher PC2 values due to larger double cone angles and more lamellae (figure 4*a*).

Centra from adult sharks occupied lower PC1 values in the morphospace, representing larger centra and juvenile sharks had smaller centra (figure 4*b*). Centra from juvenile sharks had fewer lamellae and smaller double cone angles than centra from adult sharks and occupied lower PC2 values (figure 4*b*). Using one-way ANOVAs, we found that the models examining body regions and ontogenetic status were significant for PC1 and PC2 (electronic supplementary material, table S6).

### 3.3. Landmark analysis

For the 2D geometric morphometric analysis, a Procrustes fit was used to remove influence of specimen size, position, or orientation [36,38]. We created a lollipop graph to visualize landmark variation; a point (the round ‘candy’ portion of the lollipop) portrays the average landmark location, and the direction and length of the attached line (the ‘stick’ of the lollipop) depicts the variation across all images. Additionally, we constructed a wireframe graph using 21 landmarks, which manually connects landmarks to visualize overall shape, to inspect average landmark placement across body regions. The first landmark was in the centre of each centrum, and the remaining 20 landmarks outlined the mineral plates that compose the intermedialia (figure 2*g*). Due to the Procrustes fit scaling, each specimen's landmark coordinates are superimposed into the same shape space. As a result, we do not make direct comparisons across ontogenetic stages, and we perform the wireframe assessment across body regions only [39].

**Figure 5.**
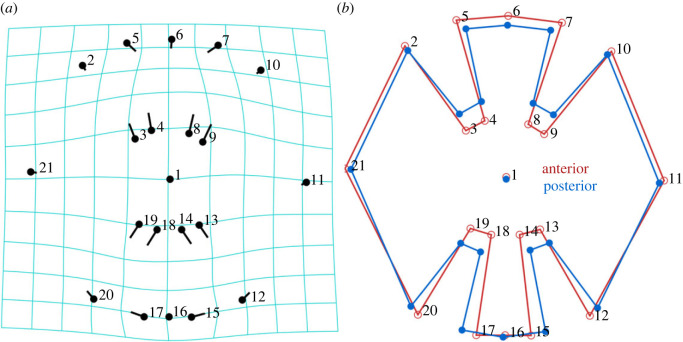
Geometric morphometric analysis. (*a*) Lollipop plot of landmarks with transformation grid displaying 3D changes in points across body regions (note the expansion in the ventral arch region, elongated ‘sticks’ of lollipops 12–20). (*b*) Wireframe graph overlaying average landmark location for an anterior body centrum (red) and posterior body centrum (blue).

The lollipop graph showed that the greatest landmark variation occurred in the eight points (3–4, 8–9, 13–14, and 18–19) proximal to the centre of the centrum and at the boundary of the mineral–arch interface (figure 5*a*). We observed that these landmarks shifted outwards in posterior body centra, shortening the gaps in the intermedialia, and increasing the mineralized area surrounding the double cone apex (figure 5*b*). Additionally, we observed a widening effect of the ventral intermedialia sector in posterior body centra (landmarks 15–17).

**Figure 6.**
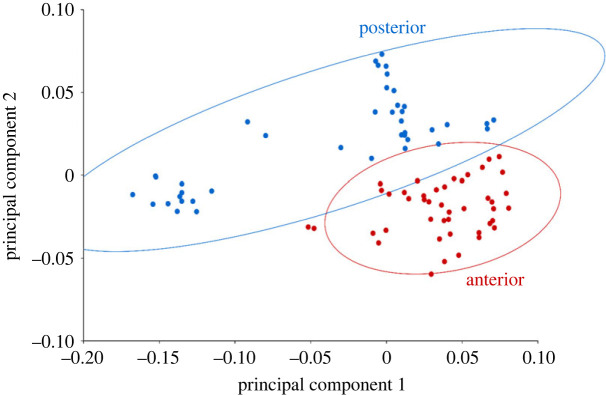
Landmark PCA across body regions. PCA coordinates were grouped by body region with 95% confidence interval ellipses. Posterior body centra had a significantly different mineral layout (quantified as shape) from anterior body centra.

We performed a PCA and Procrustes ANOVA on the landmark coordinate values and found landmarks significantly varied between body regions (*F*_38,3154_ = 30.88; *p* < 0.0001; figure 6). Posterior body centra occupied a larger range of PC1 scores than anterior body centra. On the PC2 axis, anterior body centra were dispersed across lower scores, while posterior body centra occupied higher PC2 scores.

## 4. Discussion

Our findings support a plausible hypothesis that thresher shark centra morphology and microstructure may meet mechanical demands for fast swimming and tail-whipping behaviour. We found that significant vertebral morphology and microstructure variation among body regions and across ontogeny was driven by corpus calcarea structure (double cone and intermedialia angles) and the number of lamellae. We think that the morphometrics and architecture found in anterior body vertebrae stabilize the body while precaudal pit vertebrae support the caudal fin during tail-whipping. Additionally, ontogenetic changes suggest that vertebral morphology shifts across development to support a larger body and caudal fin.

We found our two primary axes for the PCA explained 71% of the total variation and were significant across body regions and ontogeny (figure 4*a*,*b*; electronic supplementary material, table S6). The loadings suggest centra morphology, intervertebral joint shape (altered by double cone and intermedialia angles), and mineral amount are integral to the design of thresher shark vertebrae. The large double cone angles and increased mineralization (lamellae and nodes) are a distinct morphology of common thresher shark centra, and their unique tail-whipping behaviour likely drives mechanical needs of the vertebral column.

### 4.1. Vertebral structure by body region

We found that anterior body centra are wider, taller, and longer than posterior body centra, which is consistent with previous findings of common thresher shark centra (electronic supplementary material, table S1) [26,29]. Despite smaller morphometrics, posterior body centra had more lamellae but a comparable volume of mineralized cartilage compared to anterior body centra (figures 2*e* and 3*a*; electronic supplementary material, table S1). This finding suggests that the additional lamellae in posterior body centra may be an adaptation to increase the amount of mineral in that region, and support lateral body oscillations for swimming in the posterior body. Mineral amount correlates to centrum stiffness and would suggest posterior body centra are stiffer [19,20]. Previous mechanical compression experiments reported overall low stiffness of common thresher shark centra in comparison to other lamniform and carcharhiniform species [25]. However, the rate that vertebral loading occurs *in vivo* is important to consider since cartilaginous centra have viscoelastic properties [20,40]. Common thresher shark vertebrae are stiffest at faster strain rates [25]. During tail-whipping events, thresher shark centra may experience high strain rates, resulting in increased stiffness, and the increased mineral architecture (more lamellae) may support these rapid movements. The average recorded speed of tail-whip in a pelagic thresher shark (*A. pelagicus*) was 14.03 m s^−1^ with the fastest recorded speed of 21.8 m s^−1^ [8]. In comparison, the speed of the tail in the preparation phase (just prior to tail-whipping) averaged between 1 and 9 m s^−1^ [8]. The additional mineralized lamellae in posterior body centra may support the vertebral column across various loading regimes, for instance, providing a viscoelastic response during high strain tail-whipping versus swimming at cruising speed.

We found that anterior body centra had significantly higher node counts than posterior body centra (figures 2*e* and 3*d*; electronic supplementary material, table S1). Increased bifurcations in the intermedialia have been observed to increase with vertebral size in lamniform species and are thought to provide support for increases in body girth [26]. The increased bifurcations in anterior body centra may provide stability to the corpus calcarea, similar to the tesserae arrangements in elasmobranch jaw cartilage, and supportive struts for a bridge [26,41]. Furthermore, anterior body centra were longer which would increase the second moment of area in the anterior body with respect to the dorsoventral body axis used for tail-whipping [40]. Previous experiments found that the second moment of area of a centrum and centrum length were the two most important predictors for body curvature in sharks; shorter centra enable greater curvature [42]. The longer centra observed in the anterior body of common thresher sharks likely contributes to greater resistance to dorsoventral bending in the anterior region. A more stable anterior body could be beneficial to the tail-whipping behaviour of common thresher sharks considering previous kinematics research has described overhead tail-whipping behaviour as a trebuchet [8]. In support of the trebuchet hypothesis, we suggest the anterior body serves as a stiff, rotational beam and the caudal fin as a flexible sling with the tip of the tail acting as the payload. The kinetic link principle describes catapulting motion, like an overhead tail-whip, as the energy and momentum that are transferred down the body sequentially to the most distal segment [43,44]. Centra morphology and increased nodes in the anterior region may provide resistance to dorsoventral bending and efficient energy transfer for the tail-whipping behaviour of thresher sharks.

Posterior body centra had significantly smaller intermedialia angles and larger double cone angles compared to anterior body centra (figures 2*e* and 3*b*,*c*; electronic supplementary material, table S1). We found that our reported ranges for intermedialia and double cone angles were comparable to angles previously reported in thresher shark centra [15,25]. The intermedialia and double cone angles may have a substantial role in the mechanics of thresher shark vertebral columns. Within the double cone rims of consecutive vertebrae, intervertebral capsules are formed, and both centra and intervertebral joints experience strain during swimming [16]. Intervertebral capsules are hypothesized to act hydrostatically in response to external loading and may contribute to elastic energy storage of the vertebral column over successive tailbeats [16,45]. Variation in the double cone and intermedialia angles of thresher centra modifies the shape of the intervertebral capsule, and likely alters its response to loading during swimming and tail-whipping. Double cone angles were largest in common thresher shark centra when compared across species, and the significantly larger double cone angles observed in posterior body centra may form an intervertebral capsule shape ideal for extreme axial bending [15]. In bony fishes, larger intervertebral angles increase column flexibility, and the larger double cone angles observed in thresher posterior body vertebrae may act similarly by increasing flexibility in the posterior body [46]. This supports the trebuchet hypothesis for tail-whipping behaviour; smaller double cone angles in the anterior body would provide the necessary stiffness to transfer energy to the flexible posterior body enabling the caudal fin to be whipped overhead.

Dorsal arch angles were larger in posterior body centra, while ventral arch angles were larger in anterior body centra (figures 2*e* and 3*e*,*f*; electronic supplementary material, table S1). The arch angles are visualized as the gaps between sectors of intermedialia and are continuations of the dorsal and hemal arches when they are present. The cartilage cores of neural and hemal arches are not mineralized and larger arch angles would alter the distribution of the mineralized lamellae [45]. Assuming a uniform distribution and density of lamellae throughout the intermedialia, larger dorsal arch angles would reduce the number of lamellae in the dorsal half of the centrum. Fewer lamellae would decrease the overall mineral amount and likely mechanical stiffness, considering previous experiments have observed decreased stiffness in demineralized centra and centra with lower percent mineral by mass [19,20]. The gaps between intermedialia have been hypothesized to provide compliant decoupling between sectors of mineralized cartilage, and may assist centra during high amplitude bending [15]. Additionally, previous compression testing observed that the neural arch does not bear appreciable compressive loads, supporting theories that it may aid in altering stress distribution [24]. The larger dorsal arch angles observed in posterior body centra may create a pliable region surrounding the neural arch and add flexibility to the column during dorsoventral bending. During tail-whips, the dorsal half of the vertebral column would be compressed as the caudal fin is moved overhead (figure 1). The pliability of larger arch angles in the dorsal half of posterior body centra may be an adaptation for the vertebral column to withstand the extreme bending of tail-whipping.

Smaller ventral arch angles in posterior body centra may provide the opposite effect to the dorsal arch angles. By decreasing the space between intermedialia sectors, more mineralized structures (lamellae and nodes) would be present in the ventral portion of posterior body centra. The increased mineral may stiffen the ventral portion of posterior body centra, where the hemal arch and spine insert. Considering vertebrae (centra and arches) extend into the upper lobe of the caudal fin, the reinforced ventral sector of posterior body centra may provide stability to the precaudal pit and support the tail's arc during tail-whipping. We hypothesize that these shifts in unmineralized sectors (arch angles) in posterior body vertebrae allow for better compliance in the dorsal half and stability in the ventral half, allowing the precaudal pit to bend and move the tail overhead during whipping behaviors.

We found from the landmark assessment that the arrangement of mineral within centra varies significantly across body regions in common thresher sharks (figure 5). Posterior body centra have shortened gaps between intermedialia sectors and increased microstructure surrounding the double cone apex (figure 5*b*). We found larger double cone angles in posterior body centra, which has an inverse relationship with centra mechanical properties. Therefore a shift in the mineral arrangement may be an adaptation to reinforce the cone apex, while maintaining a double cone angle to optimize the hydrostatic response of the intervertebral capsule [25]. We also found a considerable expansion of the ventral intermedialia sector in posterior body centra. This shift in mineral architecture likely corresponds to the increased number of lamellae in posterior body centra likely increasing centrum stiffness. The widened ventral sector in posterior body centra may impact the insertion of the hemal arches and spine that run continuously through the elongated tail [29]. Thresher shark caudal fins are extremely flexible, and the overall shifts in mineralization of posterior body centra may provide structural support to the precaudal pit during tail-whipping [29].

### 4.2. Vertebral structure by ontogenetic status

When comparing centra between juveniles and adults, we found that centrum morphometrics and volume were smaller in juvenile sharks (electronic supplementary material, table S1). This was an expected result considering juvenile sharks are growing faster, and centrum volume has been reported to scale with increases in girth and length [26].

We found juvenile shark centra had significantly fewer lamellae than centra from adults (figures 2*e* and 3*a*; electronic supplementary material, table S1). These findings support previous observations that mineral within cartilage is continuously deposited as body size increases [18]. Additionally, shark body length, girth, and mass will all increase with size, which will impact overall swimming mechanics. Thresher shark centra may develop a greater number of lamellae with size to support the lateral oscillations necessary to propel a larger body mass during swimming. More lamellae could also be necessary for supporting the caudal fin in adult individuals. We found a positive slope when examining the CFL:FL ratio across TL indicating positive allometry where the caudal fin is proportionally larger, relative to body size, in adults than juveniles (electronic supplementary material, figure S2). This relationship suggests that the tail lengthens at a faster rate across ontogeny, and the caudal fin is nearly equal to the body size in adult sharks (CFL:FL ratio closer to 1), while the body is larger than the caudal fin in juveniles (CFL:FL ratio less than 1). Adult thresher shark centra may benefit from additional lamellae to support the caudal fin motion during tail-whipping as a catapult [8]. The kinetic link principle suggests that if the catapulted segment (the caudal fin) is lengthened but rotated at the same speed as a shorter segment, the longer segment will travel faster [41,42]. Therefore, adult thresher sharks have an advantage; they can whip a longer tail at the same speed as a juvenile shark and the longer tail will strike prey faster than a shorter tail [8]. Compared to smaller individuals, adult thresher sharks may have a greater quantity of centra lamellae to support the mechanical demands of whipping a longer tail.

Our findings of significantly higher number of nodes in adult sized individuals corroborate previous reports of increased bifurcations with increased vertebral size (figures 2*e* and 3*d*; electronic supplementary material, table S1) [26]. As previously mentioned, increased bifurcations throughout the intermedialia may offer a structural advantage for lamellae, like tesserae arrangements in jaw cartilage or supports for a bridge [26,41]. Previous research has hypothesized that bifurcations may be considered loosely controlled mineralization, in which newly mineralized tissue is deposited freely or in response to mechanical cues [15]. Our findings support this hypothesis. Centra from adult sized sharks likely experience greater mechanical demands, such as swimming or whipping forces in larger individuals, than centra from juvenile sized sharks. As a result of increased mechanical demands, centra from adult sized sharks will have more lamellae and nodes.

When examining differences between juvenile and adult sized individuals, we found no significant difference in double cone angles, but intermedialia angles were larger in adult centra (figures 2*e* and 3*b*,*c*; electronic supplementary material, tables S2 and S3). Consistent double cone angles across development may suggest the importance of the intervertebral joint shape in relation to body region rather than body size. We observed larger double cone angles in posterior body centra, which may provide more elastic energy to the posterior body through the hydrostatic response of the intervertebral capsule [16,43]. Double cone angles did not differ significantly across ontogeny which suggests that this elastic energy usage is critical to the posterior body regardless of shark size. Considering that juvenile common thresher sharks (estimated 106 cm FL) have been documented tail-whipping, double cone angle may be conserved across ontogeny due to the physical demands of supporting the tail during whipping behaviours. Larger intermedialia angles in adult centra with consistent double cone angles across ontogeny may suggest variation in the thickness of the corpus calcarea (double cone wall). In six species, cone wall thickness increases when moving radially from the centre of centra, and the common thresher had the thickest inner wall and thinnest outer wall [15]. While our study did not directly measure cone wall thickness, we hypothesize the corpus calcarea increases in thickness but at a variables rate over development. In the common thresher, the conserved cone wall thickness may provide consistent support for the intermedialia during tail-whipping. Overall, further examination into the relationship between double cone angles, intermedialia angles, and corpus calcarea thickness would enhance our understanding of shark vertebral growth and mechanics.

We found that juvenile centra had larger dorsal arch angles and adult centra had larger ventral arch angles (figures 2*e* and 3*e*,*f*; electronic supplementary material, table S1). Larger dorsal arch angles in juvenile sharks may be due to mineralization patterns across centra. For instance, a previous study examining skeletogenesis of the small-spotted catshark (*Scyliorhinus canicula*) reported the presence of neural arches in embryos while centrum calcification remained poor [46]. While skeletogenesis of catsharks may not be representative of all shark species, the mineralization pattern in common thresher sharks may be similar where neural arches are established before centra are fully mineralized. This hypothesis is supported by anecdotal evidence; we observed poor centra mineralization (indistinguishable lamellae and nodes) and apparent neural arches in the single common thresher shark embryo from this study. Considering the evident neural arches in an embryonic specimen, it is possible that dorsal arch angles decrease across ontogeny as centra increase in size and mineralization. The observed larger ventral arch angles in adult sharks may correlate with an increase in the size of the lateral processes, hemal arch, and hemal spine through ontogeny. The lateral processes of anterior body centra adduct moving caudally along the vertebral column and eventually form the hemal arch and spine in posterior body centra. Both the posterior body centra and vertebral arches extend into the upper lobe of the caudal fin and likely scale with tail growth for structural support. The increased ventral arch angles in adult sharks would allow for larger, continuous insertions of the arches along the vertebral column, and may be integral to supporting or dissipating stress of a longer caudal fin during tail-whipping.

## 5. Conclusion

We quantified the morphometrics and microstructure of centra to assess vertebral variation across body regions and ontogeny in common thresher sharks. We found that centrum morphometrics (width, height, and length) and quantity of mineral structures were the largest drivers of vertebral variation along the column and across ontogeny. Between body regions, anterior body vertebrae have a morphology and architecture significantly different from posterior body vertebrae. We hypothesize that anterior body vertebrae stabilize the main body, while vertebrae in the precaudal pit support overhead tail-whips. We found that the quantity of mineralized structures varies along the body, and the spatial distribution of the structures within centra is also a considerable factor when evaluating mineral architecture. We also found juvenile sized sharks acquire mineralized structures through ontogeny, likely to support a larger body and tail for whipping behaviours. The data presented here are an examination into vertebral morphology variations and hypothesized mechanics of extreme axial bending in thresher sharks. We suggest future studies to examine vertebrae from within the caudal fin upper lobe, as well as use 3D modelling and finite-element analysis, to enhance our understanding of tail-whipping mechanics and assess this form–function relationship.

## Data Availability

Raw micro-CT scan files are available from Morphosource (https://www.morphosource.org/projects/000583111?locale=en). Data files are available through Dryad: https://doi.org/10.5061/dryad.ksn02v79v [49]. Additional figures and tables can be found in electronic supplementary material [50].

## References

[1] Compagno LJV. 1984 FAO species catalogue. Sharks of the world. An annotated and illustrated catalogue of shark species known to date. Part 1. Hexanchiformes to Lamniformes, vol. 4, Pt 1. Rome, Italy: FAO.

[2] Compagno LJ. 2001 Sharks of the world. An annotated and illustrated catalogue of shark species known to date. Volume 2. Bullhead, mackerel, and carpet sharks (Heterodontiformes, Lamniformes, and Orectolobiformes). Rome, Italy: FAO Species Catalogue for Fishery Purposes.

[3] Ebert DA, Dando M, Fowler S. 2021 Sharks of the world: a complete guide. Princeton, NJ: Princeton University Press.

[4] Aalbers SA, Bernal D, Sepulveda CA. 2010 The functional role of the caudal fin in the feeding ecology of the common thresher shark *Alopias vulpinus*. J. Fish Biol. **76**, 1863-1868. (10.1111/j.1095-8649.2010.02616.x) 20557638

[5] Hanan DA, Holts DB, Coan AL. 1993 *California drift gill net fishery for sharks and swordfish, 1981–82 through 1990–91*. Fish Bulletin 175.

[6] Compagno LJV. 1998 Alopiidae. Thresher sharks. In FAO identification guide for fishery purposes. The living marine resources of the western central Pacific), pp. 1269-1273. Rome, Italy: FAO.

[7] Smith SE, Rasmussen RC, Ramon DA, Cailliet GM. 2008 The biology and ecology of thresher sharks (Alopiidae). In Sharks of the open ocean: biology, fisheries and conservation (eds MD Camhi, EK Pikitch, EA Babcock), pp. 60-68. Oxford, UK: Blackwell.

[8] Oliver SP, Turner JR, Gann K, Silvosa M, Jackson TD. 2013 Thresher sharks use tail-slaps as a hunting strategy. PLoS ONE **8**, e67380. (10.1371/journal.pone.0067380) 23874415 PMC3707734

[9] Allen WE. 1923 Behavior of the thresher shark. Science **58**, 31-32. (10.1126/science.58.1489.31.b) 17808324

[10] Breder CM. 1929 Certain effects in the habits of schooling fishes, as based on the observation of Jenkinsia. American Museum novitates; no. 382.

[11] Stillwell CE. 1976 Observation on the bigeye thresher shark, *Alopias superciliosus*, in the western north Atlantic. Fish Bull. **74**, 221-225.

[12] Nakano H, Matsunaga H, Okamoto H, Okazaki M. 2003 Acoustic tracking of bigeye thresher shark *Alopias superciliosus* in the eastern Pacific Ocean. Mar. Ecol. Prog. Ser. **265**, 255-261. (10.3354/meps265255)

[13] Gubanov YP. 1972 On the biology of the thresher shark [*Alopias vulpinus* (Bonnaterre)] in the northwest Indian Ocean. J. Ichthyol. **12**, 591-596.

[14] Watanabe YY, Lydersen C, Fisk AT, Kovacs KM. 2012 The slowest fish: swim speed and tail-beat frequency of Greenland sharks. J. Exp. Mar. Biol. Ecol. **426–427**, 5-11. (10.1016/j.jembe.2012.04.021)

[15] Morse PE, Stock MK, James KC, Natanson LJ, Stock SR. 2022 Shark centra microanatomy and mineral density variation studied with laboratory microcomputed tomography. J. Struct. Biol. **214**, 107831. (10.1016/j.jsb.2022.107831) 34999244

[16] Porter ME, Diaz C, Sturm JJ, Grotmol S, Summers AP, Long JH. 2014 Built for speed: strain in the cartilaginous vertebral columns of sharks. Zoology **117**, 19-27. (10.1016/j.zool.2013.10.007) 24388493

[17] Porter ME, Ewoldt RH, Long Jr JH. 2016 Automatic control: the vertebral column of dogfish sharks behaves as a continuously variable transmission with smoothly shifting functions. J. Exp. Biol. **219**, 2908-2919. (10.1242/jeb.135251) 27655825

[18] Dingerkus G, Séret B, Guilbert E. 1991 Multiple prismatic calcium phosphate layers in the jaws of present-day sharks (Chondrichthyes; Selachii). Experientia **47**, 38-40. (10.1007/BF02041246) 1999241

[19] Porter ME, Beltrán JL, Koob TJ, Summers AP. 2006 Material properties and biochemical composition of mineralized vertebral cartilage in seven elasmobranch species (Chondrichthyes). J. Exp. Biol. **209**, 2920-2928. (10.1242/jeb.02325) 16857876

[20] Porter ME, Koob TJ, Summers AP. 2007 The contribution of mineral to the material properties of vertebral cartilage from the smooth-hound shark *Mustelus californicus*. J. Exp. Biol. **210**, 3319-3327. (10.1242/jeb.006189) 17872985

[21] Ridewood WG, MacBride EW. 1921 On the calcification of the vertebral centra in sharks and rays. Phil. Trans. R. Soc. Lond. B **210**, 311-407. (10.1098/rstb.1921.0008)

[22] Dean MN, Summers AP. 2006 Mineralized cartilage in the skeleton of chondrichthyan fishes. Zoology **109**, 164-168. (10.1016/j.zool.2006.03.002) 16584875

[23] Summers AP, Long Jr JH. 2006 Skin and bones, sinew and gristle: the mechanical behavior of fish skeletal tissues. In Fish biomechanics (eds RE Shadwick, GV Lauder), pp. 141-177. Amsterdam, The Netherlands: Elsevier.

[24] Porter ME, Long Jr JH. 2010 Vertebrae in compression: mechanical behavior of arches and centra in the gray smooth-hound shark (*Mustelus californicus*). J. Morphol. **271**, 366-375. (10.1002/jmor.10803) 19862836

[25] Ingle DN, Natanson LJ, Porter ME. 2018 Mechanical behavior of shark vertebral centra at biologically relevant strains. J. Exp. Biol. **221**, jeb188318. (10.1242/jeb.188318) 30352822

[26] Natanson L, Skomal G, Hoffmann S, Porter M, Goldman K, Serra D. 2018 Age and growth of sharks: do vertebral band pairs record age? Mar. Freshw. Res. **69**, 1440-1452. (10.1071/MF17279)

[27] Seidel R, Blumer M, Chaumel J, Amini S, Dean MN. 2020 Endoskeletal mineralization in chimaera and a comparative guide to tessellated cartilage in chondrichthyan fishes (sharks, rays and chimaera). J. R. Soc. Interface **17**, 20200474. (10.1098/rsif.2020.0474) 33050779 PMC7653374

[28] Newbrey MG, Siversson M, Cook TD, Fotheringham AM, Sanchez RL. 2013 Vertebral morphology, dentition, age, growth, and ecology of the large lamniform shark *Cardabiodon ricki*. Acta Palaeontol. Polon. **60**, 877-897. (10.4202/app.2012.0047)

[29] Crofts SB, Shehata R, Flammang BE. 2019 Flexibility of heterocercal tails: what can the functional morphology of shark tails tell us about ichthyosaur swimming? Integr. Organism. Biol. **1**, obz002. (10.1093/iob/obz002) PMC7671117 33791519

[30] Natanson LJ et al. In press. Morphometric conversions for 33 shark species from the western North Atlantic Ocean. Mar. Fish Rev. **84**. (10.7755/MFR.84.3-4.1)

[31] Gervelis BJ, Natanson LJ. 2013 Age and growth of the common thresher shark in the western North Atlantic Ocean. Trans. Am. Fish. Soc. **142**, 1535-1545. (10.1080/00028487.2013.815658)

[32] Schneider CA, Rasband WS, Eliceiri KW. 2012 NIH Image to ImageJ: 25 years of image analysis. Nat. Methods **9**, 671-675. (10.1038/nmeth.2089) 22930834 PMC5554542

[33] Rohlf FJ. 2006 Tpsdig: digitize coordinates of landmarks and capture outlines. v. 2.10. Stony Brook, NY: Department of Ecology & Evolution, State University of New York.

[34] Rolfe S et al. 2021 SlicerMorph: an open and extensible platform to retrieve, visualize and analyse 3D morphology. Methods Ecol. Evol. **12**, 1816-1825. (10.1111/2041-210X.13669) PMC12094517 40401087

[35] RStudio Team. 2020 RStudio: integrated development environment for R. Boston, MA: RStudio. See http://www.rstudio.com/.

[36] Klingenberg CP. 2011 MorphoJ: an integrated software package for geometric morphometrics. Computer program note. Mol. Ecol. Resour. **11**, 353-357. (10.1111/j.1755-0998.2010.02924.x) 21429143

[37] Klingenberg CP, McIntyre GS. 1998 Geometric morphometrics of developmental instability: analyzing patterns of fluctuating asymmetry with Procrustes methods. Evolution **52**, 1363-1375. (10.2307/2411306) 28565401

[38] Dryden IL, Mardia KV. 1998 Statistical shape analysis. Wiley Series in Probability and Statistics. New York, NY: John Wiley & Sons.

[39] Hallgrimsson B, Percival CJ, Green R, Young NM, Mio W, Marcucio R. 2015 Morphometrics, 3D imaging, and craniofacial development. Curr. Top. Dev. Biol. **115**, 561-597. (10.1016/bs.ctdb.2015.09.003) 26589938 PMC5299999

[40] Wainwright SA, Gosline JM, Biggs WD, Currey JD. 1982 Mechanical design in organisms. Princeton, NJ: Princeton University Press.

[41] Summers AP, Koob TJ, Brainerd EL. 1998 Stingray jaws strut their stuff. Nature **395**, 450-451. (10.1038/26649)

[42] Porter ME, Roque CM, Long Jr JH. 2009 Turning maneuvers in sharks: predicting body curvature from axial morphology. J. Morphol. **270**, 954-965. (10.1002/jmor.10732) 19248153

[43] Fedak MA, Heglund NC, Taylor CR. 1982 Energetics and mechanics of terrestrial locomotion. II. Kinetic energy changes of the limbs and body as a function of speed and body size in birds and mammals. J. Exp. Biol. **97**, 23-40. (10.1242/jeb.97.1.23) 7086342

[44] Cross R. 2004 Physics of overarm throwing. Am. J. Phys. **72**, 305-312. (10.1119/1.1634964)

[45] Schmitz RJ. 1995 Ultrastructure and function of cellular components of the intercentral joint in the percoid vertebral column. J. Morphol. **226**, 1-24. (10.1002/jmor.1052260102) 7473764

[46] Brainerd EL, Patek SN. 1998 Vertebral column morphology, C-start curvature, and the evolution of mechanical defenses in tetraodontiform fishes. Copeia **1998**, 971-984. (10.2307/1447344)

[47] Berio F, Broyon M, Enault S, Pirot N, López-Romero FA, Debiais-Thibaud M. 2021 Diversity and evolution of mineralized skeletal tissues in chondrichthyans. Front. Ecol. Evol. **9**, 660767. (10.3389/fevo.2021.660767)

[48] Enault S, Adnet S, Debiais-Thibaud M. 2016 Skeletogenesis during the late embryonic development of the catshark *Scyliorhinus canicula* (Chondrichthyes; Neoselachii). M3 **1**, e2. (10.18563/m3.1.4.e2)

[49] Knaub JL, Passerotti M, Natanson LJ, Meredith T, Porter M. 2023 Data from: Vertebral morphology in the tail-whipping common thresher shark, *Alopias vulpinus*. Dryad Digital Repository. (10.5061/dryad.ksn02v79v) PMC10762434 38179080

[50] Knaub JL, Passerotti M, Natanson LJ, Meredith T, Porter M. 2023 Vertebral morphology in the tail-whipping common thresher shark, *Alopias vulpinus*. Figshare. (10.6084/m9.figshare.c.6984305) PMC10762434 38179080

